# Estimation of Life-Year Loss and Lifetime Costs for Different Stages of Colon Adenocarcinoma in Taiwan

**DOI:** 10.1371/journal.pone.0133755

**Published:** 2015-07-24

**Authors:** Po-Chuan Chen, Jenq-Chang Lee, Jung-Der Wang

**Affiliations:** 1 Division of Colorectal Surgery and General Surgery, Department of Surgery, National Cheng Kung University Hospital, College of Medicine, National Cheng Kung University, Tainan, Taiwan; 2 Departments of Internal Medicine and Occupational and Environmental Medicine, National Cheng Kung University Hospital, and Department of Public Health, College of Medicine, National Cheng Kung University, Tainan, Taiwan; Kaohsiung Chang Gung Memorial Hospital, TAIWAN

## Abstract

**Backgrounds and aims:**

Life-expectancy of colon cancer patients cannot be accurately answered due to the lack of both large datasets and long-term follow-ups, which impedes accurate estimation of lifetime cost to treat colon cancer patients. In this study, we applied a method to estimate life-expectancy of colon cancer patients in Taiwan and calculate the lifetime costs by different stages and age groups.

**Methods:**

A total of 17,526 cases with pathologically verified colon adenocarcinoma between 2002 and 2009 were extracted from Taiwan Cancer Registry database for analysis. All patients were followed-up until the end of 2011. Life-expectancy, expected-years-of-life-lost and lifetime costs were estimated, using a semi-parametric survival extrapolation method and borrowing information from life tables of vital statistics.

**Results:**

Patients with more advanced stages of colon cancer were generally younger and less co-morbid with major chronic diseases than those with stages I and II. The LE of stage I was not significantly different from that of the age- and sex-matched general population, whereas those of stages II, III, and IV colon cancer patients after diagnosis were 16.57±0.07, 13.35±0.07, and 4.05±0.05 years, respectively; the corresponding expected-years-of-life-lost were 1.28±0.07, 5.93±0.07 and 16.42±0.06 years, significantly shorter than the general population after accounting for lead time bias. Besides, the lifetime cost of managing stage II colon cancer patients would be US $8,416±1939, 14,334±1,755, and 21,837±1,698, respectively, indicating a big saving for early diagnosis and treatment after stratification for age and sex.

**Conclusions:**

Treating colon cancer at younger age and earlier stage saves more life-years and healthcare costs. Future studies are indicated to apply these quantitative results into the cost-effectiveness evaluation of screening program for colon cancers.

## Introduction

For years, researchers have attempted to extrapolate the cancer survival rates using parametric models, such as the Weibull distribution[1], exponential distribution[2], Gompertz log-normal distribution[3], and extrapolation technique[4]; however, results are usually suboptimal, particularly for cancers with high-censored rates[5]. To identify a solution for this problem, our group developed a semiparametric method since 1999 [6–8]. We incorporated the survival functions of age- and sex-matched referents from the general population in the estimation process and assumed that patients with a specific type of cancer would die prematurely, which represented a cancer-associated “constant excess hazard”[6]. The idea of cancer-associated “constant excess hazard” has been demonstrated statistically both by our group and by another study group in Sweden[9]. Using this method, the life-expectancy (LE) of a cancer cohort after diagnosis can be extrapolated with acceptable accuracy. In addition, this method can be used to compute the expected-years-of-life-lost (EYLL) that would have been saved had the patient not developed cancer[10].

Since 2006, the members of our research group have already been using this method to measure the LE and EYLL in different clinical scenarios [11–14]. In this report, we present the results of estimating the LE and EYLL of colon cancer patients in Taiwan using our semiparametric method and a nation-wide, population-based cancer registry database from Taiwan.

## Materials and Methods

### Cohort establishment and datasets

Since the Taiwan Cancer Registry (a national-scale, population-based cancer registry) was founded in 1979, hospitals that provide both outpatient and hospitalized cancer care and have a greater than 50-bed capacity have been recruited to participate in reporting all newly-diagnosed malignant neoplasms. The registry has been organized and funded by the Ministry of Health and Welfare of the Executive Yuan of the Taiwan government. The National Public Health Association has been contracted to operate the registry, and it has organized an advisory board to standardize definitions of terminology, coding, and procedures throughout the registry’s reporting system [15]. On the other hand, the National Health Insurance (NHI), launched in 1995, is a compulsory program with 99.9% of coverage in 2013 and is responsible for virtually all the healthcare reimbursement plans in Taiwan.

Our study was approved by the internal review board of the National Vital Statistics. All the patient data were anonymized and de-identified prior to analysis. Using the International Classification of Diseases for Oncology, 3rd Edition (ICD-O-3) coding, the following site codes were included: c180, c182-187, and c199, which represented all colon cancers with exact primary sites. Scanning with these site codes, a total of 37,419 colon cancer cases occurring between 2002 and 2009 were identified from the Taiwan Cancer Registry database. We deliberately included only primary cases with a definite pathology of adenocarcinoma (morphology code: 814) and complete staging data, while excluded cases without a pathology report, and those of carcinoma in situ, double or multiple cancers. In the end, a total of 17,526 cases were eligible for further analysis. All patients were followed-up until the end of 2011 by linkage with the National Mortality Registry of Taiwan. They were abstracted for co-morbidities with major chronic diseases within three years prior to the diagnosis of colon cancer, including diabetes mellitus, hypertension, stroke, acute myocardial infarction, end-stage renal disease, and chronic obstructive lung disease. To analyze the lifetime cost of all cohort patients, we extracted the reimbursement data files from the NHIRD (National Health Insurance Research Database) of Taiwan, plus adjustment of 2010 CPI (consumer price index). These files contained detailed demographic data and information regarding the healthcare services provided for each patient, including all payments for outpatient visits, hospitalizations, prescriptions, and intervention procedures. Because cancer patients with a pathological diagnosis are eligible for waiving all co-payments in the NHI of Taiwan, our data are generally very comprehensive.

**Fig 1:**
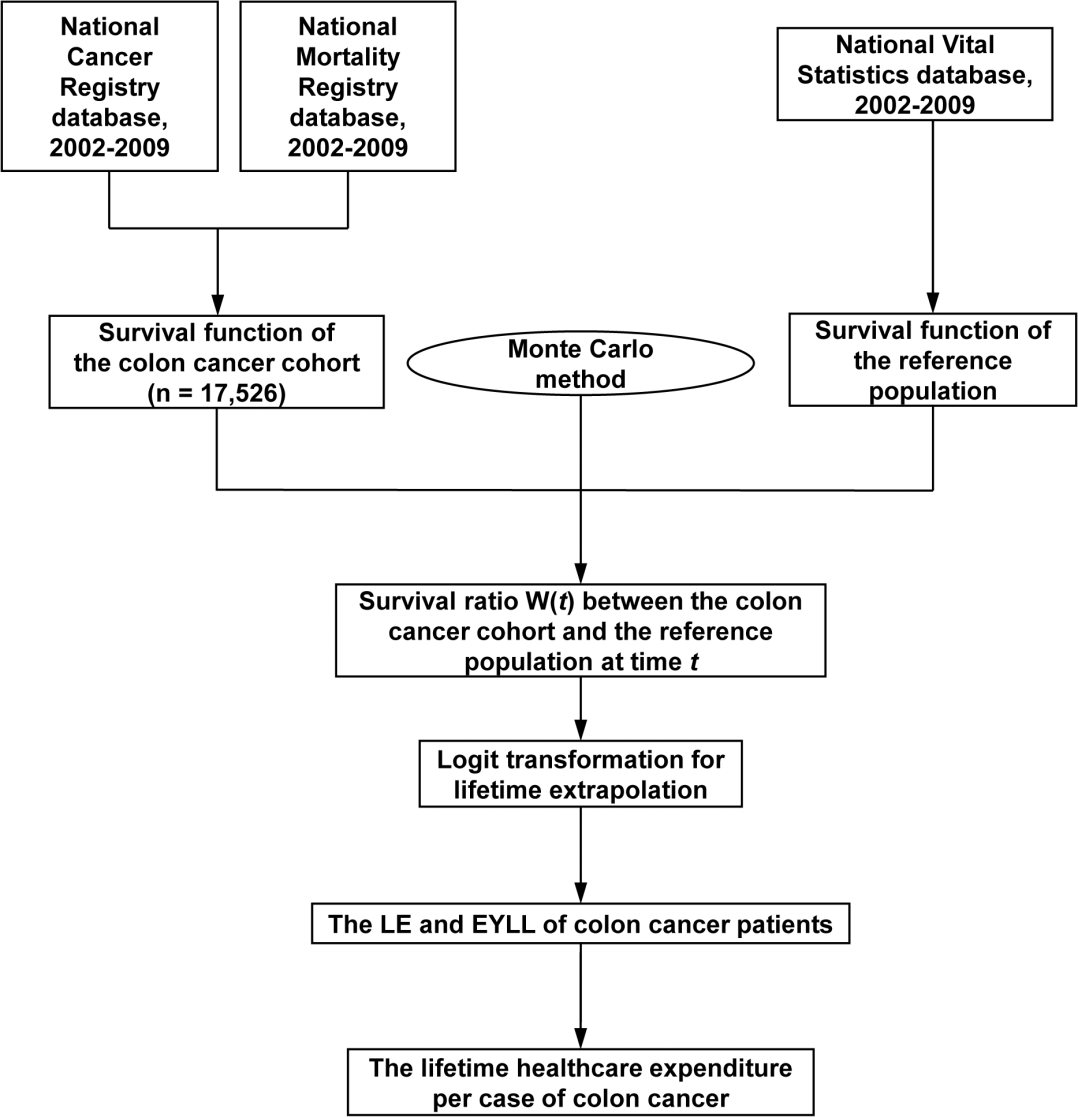
Flow chart of how the data were collected and semiparametric survival extrapolation method was conducted. (LE: life expectancy, EYLL: expected years of life loss)

A survival extrapolation method that was created and validated by our study group was applied to estimate the lifetime survival function of this patient cohort [11–14]. The algorithm of how we proceeded is summarized in Fig 1 and as follows:

### Lifetime survival extrapolation and validation

All of the above patients were followed until the end of 2011 and linked with the National Mortality Registry to obtain the survival function via the Kaplan-Meier estimation method. On the other hand, we extrapolated the survival function to lifetime by assuming a “constant excess hazard” for colon cancer patients. The procedure could be summarized in the following three steps: First, we took the hazard functions from the life tables of the National Vital Statistics of Taiwan to generate an age- and sex-matched reference population using the Monte Carlo method. The survival functions of this reference population were estimated objectively, thereby acting as a yardstick. Second, we calculated the survival ratio, denoted as W(*t*), between the cancer cohort and the reference population at each time *t* period, which could be expressed as $\text{W}\left(t\right)\;=\frac{\text{S}\;(t,\text{cancer cohort})}{\text{S}\;(t,\text{reference population})}$

Theoretically, because the cancer cohort had a worse survival than the reference population, the value of W(*t*), initially equals 1, would gradually decrease to 0 due to cancer-associated excess mortality. To facilitate extrapolation, we used the logit transformation of W(*t*) for linear regression analysis. If the cancer-associated excess hazard remained constant over time, the curve of the logit of W(*t*) would gradually converge to a straight line, indicating the existence of “constant excess hazard”. Third, the slope of the estimated straight regression line, together with the survival functions of the reference population beyond the follow-up limit, was used to extrapolate the lifetime survival functions of the colon cancer cohort. In this way, the LE of the colon cancer cohort (with extrapolation up to 600 months or 100 years old) after diagnosis was estimated. The EYLL of the colon cancer cohort was defined as the lifetime survival difference between the cohort and the reference population; in other words, the loss of LE.

The semi-parametric survival extrapolation method described above had already been demonstrated using computer simulation and had been proven mathematically by our group [5,6]. An open-access iSQoL statistical package was used to facilitate the computation (http://www.stat.sinica.edu.tw/isqol/). To validate the survival extrapolation method, we used the survival data from patients who were diagnosed during the first 5 years and extrapolated these up to 10 years using the method described above. Because these patients were actually followed clinically until the end of 2011, the mean survival period within the 10-year follow-up period, which was estimated using the Kaplan–Meier method, could be considered as our gold standard metric. Relative biases were computed to compare the difference in values between the extrapolation and the Kaplan–Meier estimation.

The calculation of lifetime reimbursement costs were as follows: We summed the monthly expenditures for all patients, including the cost of inpatient, outpatient and emergency costs for the treatment of colon cancer after diagnosis, and divided total amounts by the number of these patients during each month to estimate the monthly average costs to the NHI. NHI expenditures were first adjusted to the 2010 CPI and exchange rate (1USD = 29.322 TWDs), followed by applying a 3% annual discount rate. The monetary value after the end of the follow-up period was assumed to be the same as the average of the last 10% of measurements through smoothing to extrapolate lifetime costs. The total average monthly expenditures were multiplied by the monthly survival probabilities for each stage and age group over the course of a lifetime, and all these monetary values were summed to obtain the lifetime healthcare expenditure for each group.

### Statistical analysis

The survival function was calculated using the Kaplan–Meier method and was then extrapolated to lifetime, the confidence limit of which was estimated using the bootstrap method by repeating sampling 100 times. The differences between groups were assessed using the Z-test, and p < 0.05 was considered statistically significant.

## Results

In general, our patients of colon adenocarcinoma are of middle and old age and many of them suffered from other common chronic diseases, including diabetes, hypertension, etc. As summarized in the Table 1, patients with more advanced stages were generally younger than those with stages I and II, and there is no linear trend for association between advanced stages and increased co-morbidities. In fact, there appears a reversed trend of fewer co-morbidities in all major chronic diseases among patients with more advanced stage of colon adenocarcinoma.

**Table 1:** Demographic and clinical characteristics of patients with colon adenocarcinoma.

	Stage Ⅰ	Stage Ⅱ	Stage Ⅲ	Stage Ⅳ	p-value
**Total no. of cases (%)**	2076 (11.85%)	5917 (33.76%)	6012 (34.30%)	3521 (20.09%)	<0.0001
**Mean age (S.D.)**	65.29 (12.58)	66.60 (13.34)	64.61 (13.52)	62.99 (13.91)	<0.0001
**% males**	55.35%	55.43%	53.16%	53.22%	0.0336
**Tumor site**					<0.0001
Right side colon^a^ (%)	636 (30.64%)	2439 (41.22%)	2247 (37.38%)	1406 (39.93%)	
Left side colon^b^ (%)	1440 (69.36%)	3478 (58.78%)	3765 (62.62%)	2115 (60.07%)	
**Co-morbidities**					
Diabetes mellitus (%)	273 (13.15%)	764 (12.91%)	709 (11.79)	373 (10.59%)	0.0031
Hypertension (%)	480 (23.12%)	1341 (22.66%)	1214 (20.19%)	605 (17.18%)	<0.0001
Stroke (%)	156 (7.51%)	526 (8.89%)	441 (7.34%)	215 (6.11%)	<0.0001
Myocardial infarction (%)	32 (1.54%)	89 (1.50%)	88 (1.46%)	35 (0.99%)	0.1600
End stage renal disease (%)	37 (1.78%)	91 (1.54%)	69 (1.15%)	34 (0.97%)	0.0159
Chronic obstructive lung diseases (%)	60 (2.89%)	134 (2.26%)	130 (2.16%)	54 (1.53%)	0.0071

^a^ICD-9 codes 180, 182, 183, 184.

^b^ICD-9 codes 185,186,187,199.

**Fig 2:**
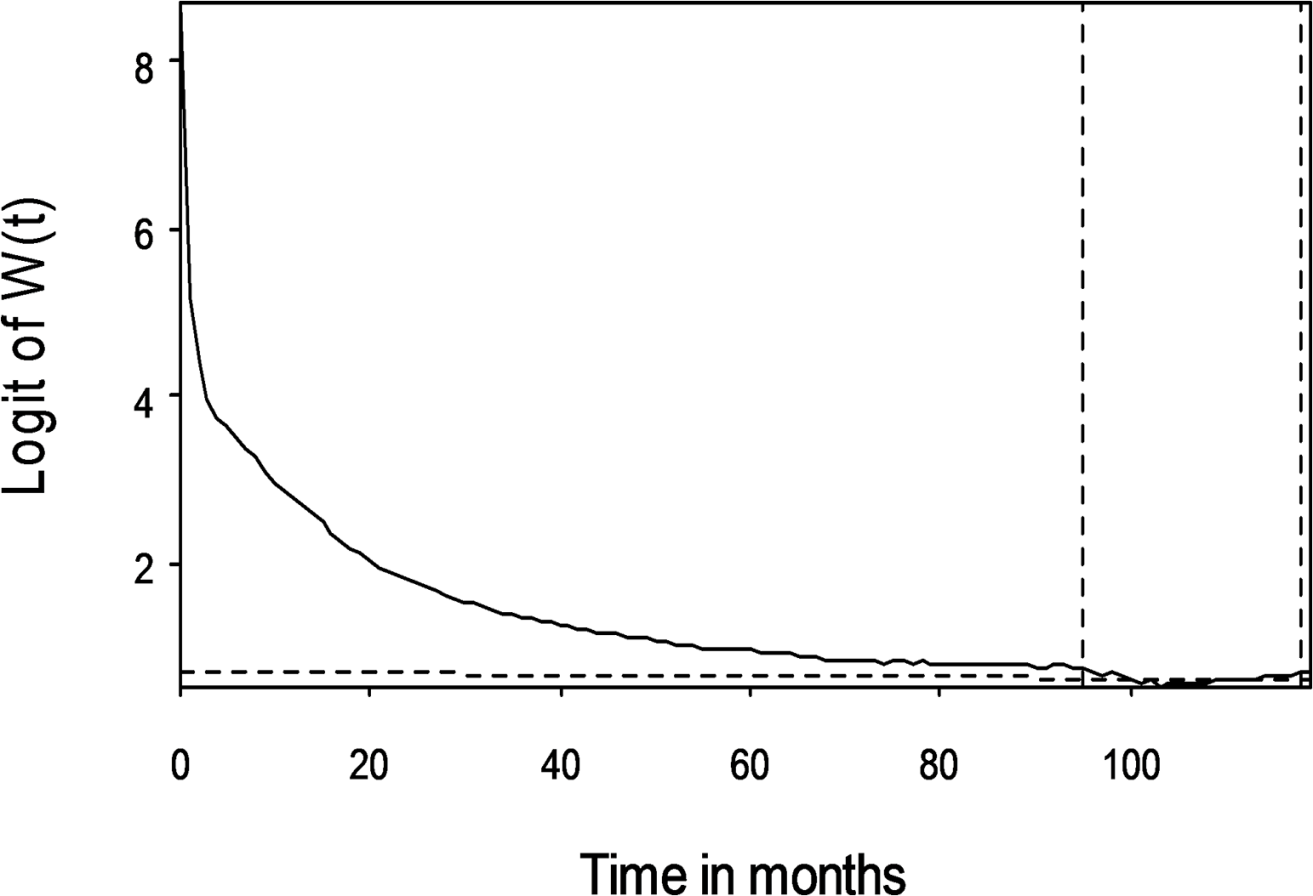
The logit of W(*t*) in stage III colon cancer patients. The extrapolation period was located between 95 and 118 months after diagnosis.

The logit of W(*t*) of stage III colon cancer cohort, as an example, was expressed in Fig 2, showing the fulfillment of cancer-associated “constant excess hazard” assumption. In our colon cancer cohort, this phenomenon could be seen in all but stage I patients, of which no survival inferiority could be demonstrated during logit transformation, indicating that stage I colon cancer patient had no worse prognosis than the general population.

**Fig 3:**
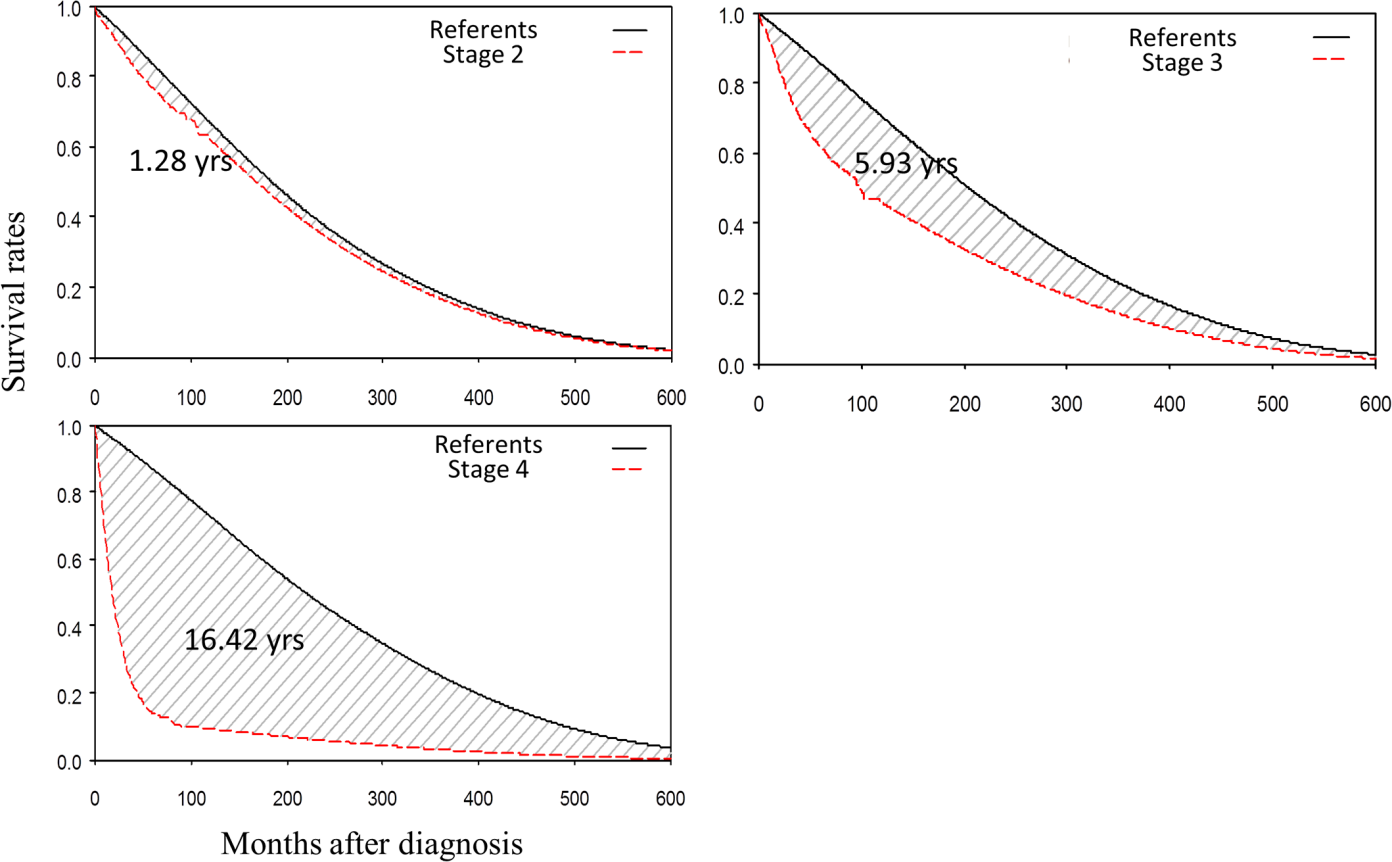
Average expected years of life lost (EYLL) of colon cancer patients by different stages. The differences of LE between colon cancer patients and the sex- and age-matched referents were represented in the shadowed area.

In Taiwan, five-year survival rates of stageⅠto IV after colon cancer diagnosis were 87.79%, 76.79%, 62.24% and 14.17%, respectively, while those of ten-year rates were 66.80%, 63.23%, 47.11% and 10%, respectively (S1 Fig). Our result indicated that, for colon cancer patients, treatment outcomes in Taiwan were comparable to those of the western countries. Concerning survival extrapolation, the corresponding LE’s of stage II, III and IV colon cancer patients were 16.57, 13.35 and 4.05 years; those of EYLL’s were 1.28, 5.93 and 16.42 years, respectively (Table 2 and Fig 3). For stages II~IV, the differences of LE between different stages and different ages or gender groups were all statistically significant (Tables 2 and 3). The point that logit of W(t) started to approach zero, meaning that colon cancer patients started to have no inferior survival than the sex- and gender-matched general population, started at 65, 71, and 82 months respectively after the initial diagnosis for stage II, III and IV colon cancer patients (data not shown). In general, the younger the age of diagnosis and/or the earlier the stage, the higher the LE and EYLL, which were simultaneously, accompanied with lower lifetime expenditures. Female patients had higher EYLL’s, probably because female residents in Taiwan have a longer LE than that of the male residents.

**Table 2:** The life expectancy (LE), expected years of life lost (EYLL), and lifetime costs of colon adenocarcinoma by stage and gender.

Stage	SEX	Case Number	Age (SD^a^)	LE (SE^b^)	EYLL (SE^b^)	Lifetime costs (USD)
**Ⅰ**	All	2076	65.29 (12.58)			
	M	1149	66.19 (12.36)			
	F	927	64.17 (12.77)			
**II**	All	5917	66.6 (13.34)	16.57 (0.07)	1.28 (0.07)	8416±1939
	M	3280	66.86 (13.02)	15.29 (0.05)	1.17 (0.04)	8661±1776
	F	2637	66.27 (13.71)	16.58 (0.07)	2.83 (0.07)	8201±1766
**III**	All	6012	64.61 (13.52)	13.35 (0.07)	5.93 (0.07)	14334±1755
	M	3196	65 (13.37)	12.72 (0.06)	5.05 (0.06)	15011±1959
	F	2816	64.17 (13.68)	13.66 (0.09)	7.45 (0.09)	13657±1693
**IV**	All	3521	62.99 (13.91)	4.05 (0.05)	16.42 (0.06)	21837±1698
	M	1874	64.29 (13.27)	3.9 (0.06)	14.32 (0.07)	22661±1669
	F	1647	61.52 (14.46)	3.29 (0.06)	19.86 (0.06)	21166±1435

^a^SD: Standard deviation.

^b^SE: Standard error.

**Table 3:** The life expectancy (LE), expected years of life lost (EYLL), and lifetime costs of colon adenocarcinoma by stage and ages.

Stage	Age	Case Number	Age (SD^a^)	LE (SE^b^)	EYLL (SE^b^)	Lifetime costs (USD)
**II**	<50	682	41.88 (6.4)	34.38 (0.08)	3.57 (0.08)	9762±1598
	50–64	1641	57.46 (4.23)	21.41 (0.07)	2.76 (0.07)	9314±1518
	≧65	3594	75.47 (6.64)	10.29 (0.05)	0.92 (0.04)	7862±1934
**III**	<50	819	41.49 (6.26)	25.69 (0.1)	12.68 (0.11)	20197±2075
	50–64	1974	57.23 (4.23)	17.4 (0.1)	7.11 (0.1)	16762±1758
	≧65	3219	75.02 (6.54)	7.99 (0.09)	3.6 (0.09)	12057±1745
**IV**	<50	614	41.33 (6.52)	3.25 (0.1)	35.66 (0.1)	29712±1777
	50–64	1163	57.02 (4.22)	3.97 (0.09)	20.75 (0.1)	25365±1482
	≧65	1744	74.6 (6.31)	2.83 (0.06)	8.92 (0.06)	16485±1824

^a^SD: Standard deviation.

^b^SE: Standard error.

To validate our extrapolation method, we provided the extrapolation results using first 5-year follow-up data to extrapolate the next 5-year period and made a comparison with the exact 10-year survival based on Kaplan-Meier estimates (Table 4). All the results showed relative biases of less than 10% except for one prediction with an actual follow-up value of 2.53 years (stage IV patients with short LE), with which a small difference of -0.37 years in survival estimate could result in -14.78% of relative bias in value. Besides, most of these biases showed negative values, indicating an underestimation of survival.

**Table 4:** Estimates of mean survival years in 10-year of follow-up based on the first 5-year of follow-up data and compared with Kaplan-Meier (K-M) estimates of 10 years of follow-up.

Stage	Age	Cohort size	Age at diagnosis (SD^a^)	Censored rate (%)	10-year survival based on K-M estimate (SE^b^) (month)	Extrapolation based on first 5-year follow up (SE^b^) (month)	Relative bias^c^ (%)
**II**	<50	327	41.35 (6.62)	96.02	9.01 (0.14)	9.18 (0.04)	1.88
	50–64	748	57.56 (4.39)	95.72	8.67 (0.12)	8.05 (0.02)	-7.23
	≧65	1567	74.99 (6.52)	87.75	6.88 (0.11)	7.03 (0.05)	2.30
**III**	<50	381	41.36 (6.42)	86.09	7.15 (0.17)	6.5 (0.12)	-9.00
	50–64	852	57.47 (4.34)	88.85	7.24 (0.13)	6.88 (0.12)	-5.09
	≧65	1346	74.75 (6.36)	78.53	5.6 (0.12)	5.39 (0.04)	-3.91
**IV**	<50	294	41.46 (6.86)	49.32	2.26 (0.14)	2.42 (0.12)	7.39
	50–64	474	57 (4.24)	56.96	2.53 (0.12)	2.16 (0.06)	-14.78
	≧65	719	74.28 (6.01)	43.53	2.12 (0.09)	1.95 (0.07)	-8.08

^a^SD: Standard deviation.

^b^SE: Standard error.

^c^Relative bias = (Estimate from extrapolation–K-M estimate)/ K-M estimate.

The lifetime cost generally increased with more advanced cancer stages. This study showed that managing a patient with stage II colon cancer costs US $8,416 with an LE of 16.57 years, whereas managing a patient with stage IV colon cancer cost US $21,837 with an LE of 4.05 years, indicating a big saving for early diagnosis and treatment. Comparatively, the cost-per-LY (life year) of treating stage II colon cancer (= US $508 with no adjustment of discount rate over survival) would be much lower than that of the stage IV colon cancer (= US $5392, same as above). When taking age factor into consideration, the lifetime cost of treating stage II colon cancer with less than 50 years of age would be the lowest.

## Discussion

In this retrospective, population-based study, we showed unequivocal evidence for the savings of life years and lifetime costs when treating early stages and younger patients with colon adenocarcinoma. Besides, we demonstrated that using a nation-scale database, LE and EYLL of colon cancer patients could be accurately estimated by different stages and age groups.

We also provide following arguments to corroborate the above assertion: First, since our patients with advanced stages (III and IV) of colon adenocarcinoma were generally younger and less co-morbid with major chronic diseases (Table 1), their loss of life expectancy could mainly be attributed to the advanced stages. Second, all survival functions of stages II-IV colon cancer were demonstrated to fulfill the assumption of a cancer-related “constant excess hazard” (Fig 2 as an example)[5,12], which indicate the valid estimations of the LE and EYLL for these patients. Third, in general, the longer the follow-up, the higher the accuracy of estimating the life expectancy since the slope of logit of W(*t*) would converge and be more precisely estimable. Given the results of validation test of applying the same method on the first 5 years to predict the end of 10-year (Table 4), we believe that our final estimation of long term survival, based on 10 years of follow-up, would be more accurate and the potential bias would be less than 10%. This fact was also demonstrated by the Swedish group that, using Swedish Cancer Registry with 40 years of follow-up, very accurate survival extrapolation could be achieved by a method very similar to ours[9].

Fourth, since we have included all patients with staging information of colon adenocarcinoma in Taiwan during 2002–2009, this study could be considered as representative of the outcome of our current practice of the last decade. Although our outcomes appear slightly better, we have corroborated the results of LE stratified by stages from another population-based study of southern Netherlands, of which they compiled the long term life table to estimate the LE for patients with colorectal cancer [16,17]. Another study from Italy[18] also confirmed our estimation, in which study they showed that in general, colorectal cancer patients who received standard treatment could be considered as almost “cured” about 8–9 years after the initial diagnosis. Their finding is comparable to our findings in the slope of logit of W(*t*), in which more than 5, 6 and 7 years of inferior over-all survival could be expected for stage II, III and IV colon cancer patients, respectively. Fifth, we have shown that the EYLL’s for different stages also show the same trend. The EYLL represents the expected life-years that could be saved, had patients not developed the cancer. Since it is already adjusted for lead time bias, it would be a more accurate indicator for outcome comparison for different stages. Thus, we tentatively concluded that diagnosing and treating patients as early as possible would save more lives and more healthcare costs.

Our study can be applied to several dimensions: First, since detecting and treating a colon cancer before reaching stage IV would be saving life-years and costs, we should promote screening and early detection of colon cancer among asymptomatic people. To advocate such a screening program, we could inform them that there is always potential savings of life-years and possible out-of-pocket money as long as they are detected during premalignant stage and stages 0-III, and the earlier, the better. However, at what age should such a program begin depends on the costs of screening and diagnostic workup program, incidence rates of different ages, length of detectable preclinical phase, which deserve more studies in the future. Second, instead of waiting for several decades to construct the life table for patients with colon cancer [16,17], our method provides an easy and ready alternative for patients and physicians to quickly capture the LE with reasonable accuracy. Besides, it also provides estimates of EYLL, which reduces the lead time bias and would be more suitable for a fair comparison of long-term survival outcome of cancer patients with different ages. But future studies are warranted to incorporate measurements of patient reported outcome with lifetime survival function for a more comprehensive evaluation of quality of care for colon cancer patients. Third, our result also shows a stark contrast to the conventional way of presenting prognosis and raises the issue that both concepts of the LE and EYLL should be incorporated into clinical practice and patient communication. Conventionally, the prognosis of a specific type of cancer is presented as the 5-year overall or relative survival rates[19]. Our study attempts to answer the same question but with a different perspective. We begin from the date of diagnosis and estimate the lifetime survival functions, LE, and EYLL. With these results, a colorectal surgeon or physician can confidently inform a patient with newly-diagnosed colon cancer of both the general 5-year survival rates and his/her LE and EYLL, corresponding to his/her age and stage at diagnosis.

Our study has several limitations. First, this study did not incorporate information about results of currently available screening program into the analysis, and we were unable to make any more inference on outcome directly related to screening programs. More studies will be needed to clarify the cost-effectiveness of such practices. Second, since people in different countries generally have different incomes, insurance systems, and consumer prices, the lifetime costs cannot be directly generalizable to other countries. The lack of data of out-of-pocket payment, including at least transportation cost, manpower required to care for the patients, etc., would generally under-estimate the total costs to the society. However, as our NHI waives all co-payments of pathologically verified colon cancer, the relative lifetime costs of different stages would be useful to other countries for promotion of colon cancer screening. Third, as we were unable to quantify the exact lifetime survival function for stage I patients, this study did not provide an estimation of lifetime healthcare costs for it. However, since the standard guidelines for treating stage I colon cancer is surgical intervention followed by long term follow-up, the lifetime cost would generally be smaller than all the other stages.

## Conclusion

Using our survival extrapolation method, the LE and EYLL of colon cancer patients can be accurately estimated by using a nation-scale, population-based database. Our result shows that treating patients with colon adenocarcinoma at younger age and earlier stage saves more life-years and healthcare expenditures: the earlier, the better, which implies the importance of early detection and treatment. Future studies are indicated to count the loss of out-of-pocket money and apply these quantitative results into the cost-effectiveness evaluation of screening program for colon cancers.

## Supporting Information

S1 Fig Survival rates of stage I–IV colon cancer patients. Five-year survival rates were 87.79%, 76.79%, 62.24% and 14.17%, respectively; ten-year rates were 66.80%, 63.23%, 47.11% and 10%, respectively.
(TIF)
