# Supplementary figures and images for: Estimation of Life-Year Loss and Lifetime Costs for Different Stages of Colon Adenocarcinoma in Taiwan
Source: PLoS One. 2015 Jul 24;10(7):e0133755. doi: 10.1371/journal.pone.0133755 (PMC4514792; doi:10.1371/journal.pone.0133755)

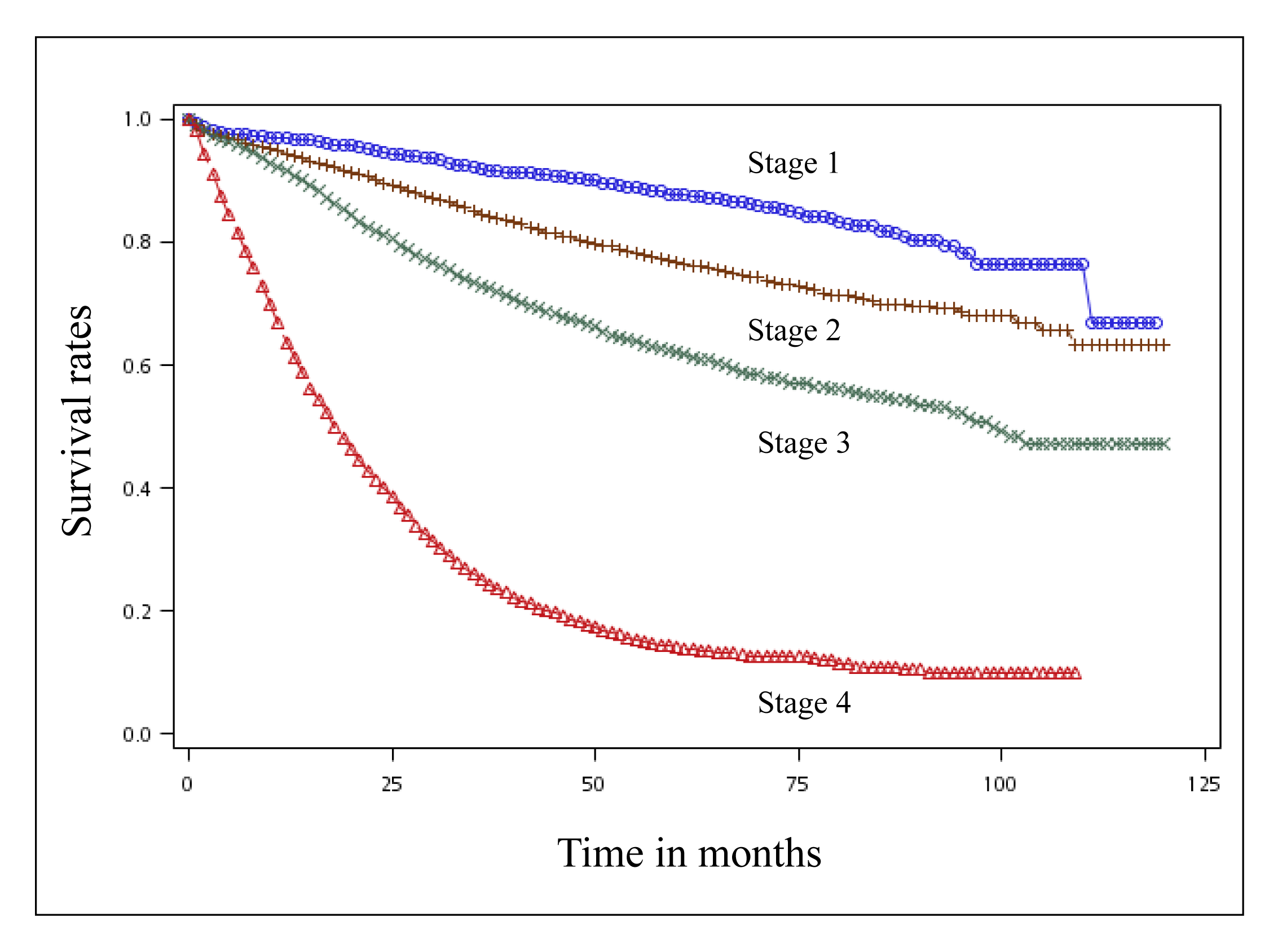

Supplement: S1 Fig — Five-year survival rates were 87.79%, 76.79%, 62.24% and 14.17%, respectively; ten-year rates were 66.80%, 63.23%, 47.11% and 10%, respectively. (TIF) [file pone.0133755.s001.tif]
